# The Level and Nature of Autistic Intelligence II: What about Asperger Syndrome?

**DOI:** 10.1371/journal.pone.0025372

**Published:** 2011-09-28

**Authors:** Isabelle Soulières, Michelle Dawson, Morton Ann Gernsbacher, Laurent Mottron

**Affiliations:** 1 Centre d'Excellence en Troubles Envahissants du Développement de l'Université de Montréal (CETEDUM), Montréal, Québec, Canada; 2 Department of Psychiatry, Massachusetts General Hospital, Harvard Medical School, Boston, Massachusetts, United States of America; 3 Department of Psychology, University of Wisconsin-Madison, Madison, Wisconsin, United States of America; Alexander Flemming Biomedical Sciences Research Center, Greece

## Abstract

A distinctively uneven profile of intelligence is a feature of the autistic spectrum. Within the spectrum, Asperger individuals differ from autistics in their early speech development and in being less likely to be characterized by visuospatial peaks. While different specific strengths characterize different autistic spectrum subgroups, all such peaks of ability have been interpreted as deficits: isolated, aberrant, and irreconcilable with real human intelligence. This view has recently been challenged by findings of autistic strengths in performance on Raven's Progressive Matrices (RPM), an important marker of general and fluid intelligence. We investigated whether these findings extend to Asperger syndrome, an autistic spectrum subgroup characterized by verbal peaks of ability, and whether the cognitive mechanisms underlying autistic and Asperger RPM performance differ. Thirty-two Asperger adults displayed a significant advantage on RPM over Wechsler Full-Scale and Performance scores relative to their typical controls, while in 25 Asperger children an RPM advantage was found over Wechsler Performance scores only. As previously found with autistics, Asperger children and adults achieved RPM scores at a level reflecting their Wechsler peaks of ability. Therefore, strengths in RPM performance span the autistic spectrum and imply a common mechanism advantageously applied to different facets of cognition. Autistic spectrum intelligence is atypical, but also genuine, general, and underestimated.

## Introduction

Individuals on the autistic spectrum are currently identified according to overt atypicalities in socio-communicative interactions, focused interests and repetitive behaviors [1]. More fundamentally, individuals on the autistic spectrum are characterized by atypical information processing across domains (social, non-social, language) and modalities (auditory, visual), raising the question of how best to assess and understand these individuals' intellectual abilities. Early descriptions [2], [3] and quantifications (e.g. [4]) of their intelligence emphasized the distinctive unevenness of their abilities. While their unusual profile of performance on popular intelligence test batteries remains a durable empirical finding [5], it is eclipsed by a wide range of speculative deficit-based interpretations. Findings of strong performance on specific tests have been regarded as aberrant islets of ability arising from an array of speculated deficits (e.g., “weak central coherence”; [6]) and as incompatible with genuine human intelligence. For example, Hobson ([7], p. 211) concluded that regardless of strong measured abilities in some areas, autistics lack “both the grounding and the mental flexibility for intelligent thought.”

Thus, there is a long-standing assumption that a vast majority of autistic individuals are intellectually impaired. In recent years, this assumption has been challenged by investigations that exploit two divergent approaches —represented by Wechsler scales of intelligence and Raven's Progressive Matrices— to measuring human intelligence [8]. Wechsler scales estimate IQ through batteries of ten or more different subtests, each of which involves different specific oral instructions and tests different specific skills. The subtests are chosen to produce scores that, for the typical population, are correlated and combine to reflect a general underlying ability. Advantages of this approach include the availability of subtest profiles of specific skill strengths and weaknesses, index scores combining related subtests, and dichotomized Performance versus Verbal IQ scores (PIQ vs. VIQ), as well as a Full-Scale IQ (FSIQ) score. However, the range of specific skills assayed by Wechsler scales is limited (e.g., reading abilities are not included), and atypical individuals who lack specific skills (e.g., typical speech processing or speech production) or experiences (e.g., typical range of interests) may produce scores that do not reflect those individuals' general intelligence.

In contrast, Raven's Progressive Matrices (RPM) is a single self-paced test that minimizes spoken instruction and obviates speech production or typicality of experiences [9]. The format is a matrix of geometric designs in which the final missing piece must be selected from among an array of displayed choices. Sixty items are divided into five sets that increase progressively in difficulty and complexity, from simple figural to complex analytic items. RPM is regarded both as the most complex and general single test of intelligence [10], [11] and as the best marker for *fluid* intelligence, which in turn encompasses reasoning and novel problem-solving abilities [8], [12]. RPM tests flexible co-ordination of attentional control, working memory, rule inference and integration, high-level abstraction, and goal-hierarchy management [13], . These abilities, as well as fluid intelligence itself, have been proposed as areas of deficit in autistic persons, particularly when demands increase in complexity [16], [17], [18], [19].

**Figure 1:**
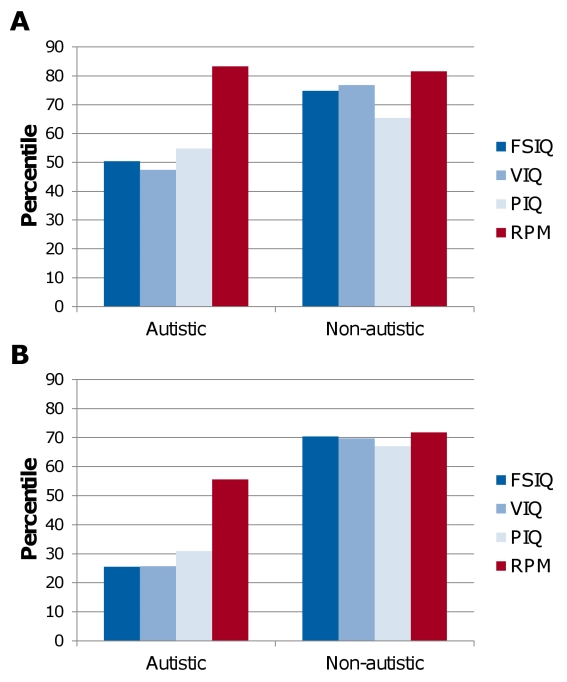
Performance on the Wechsler Intelligence Scales and Raven's Progressive Matrices by autistic and non-autistic adults (A) and children (B). Adapted from Dawson et al., 2007.

Against these assumptions, we reported that autistic children and adults, with Wechsler FSIQ ranging from 40 to 125, score an average 30 percentile points higher on RPM than on Wechsler scales, while typical individuals do not display this discrepancy, as shown in Figure 1 [20]. RPM item difficulty, as reflected in per-item error rate, was highly correlated between the autistic and non-autistic children (*r* = .96). An RPM advantage for autistic individuals has been reported in diverse samples. Bolte et al. [21] tested autistic, other atypical (non-autism diagnoses), and typical participants who varied widely in their age and the version of Wechsler and RPM they were administered; autistics with Wechsler FSIQ under 85 were unique in having a relative advantage on RPM. Charman et al. [22] reported significantly higher RPM than Wechsler scores (FSIQ and PIQ) for a large population-based sample of school-aged autistic spectrum children. In Morsanyi and Holyoak [23], autistic children, who were matched with non-autistic controls on two Wechsler subtests (Block Design and Vocabulary), displayed a numeric, though not significant, advantage within the first set of Raven's Advanced Progressive Matrices items.

The nature of autistic intelligence was also investigated in an fMRI study [24]. Autistics and non-autistics matched on Wechsler FSIQ were equally accurate in solving the 60 RPM items presented in random order, but autistics performed dramatically faster than their controls. This advantage, which was not found in a simple perceptual control task, ranged from 23% for easier RPM items to 42% for complex analytic RPM items. Autistics' RPM task performance was associated with greater recruitment of extrastriate areas and lesser recruitment of lateral prefrontal and medial posterior parietal cortex, illustrating their hallmark enhanced perception [25]. One replicated manifestation of autistics' enhanced perception is superior performance on the Wechsler Block Design subtest, suggesting a *visuospatial peak* of ability [26]. Even when autistics' scores on all other Wechsler subtests fall below their RPM scores, their Block Design and RPM scores lie at an equivalent level [20]. Thus, enhanced occipital activity, superior behavioral performance on RPM, and visuospatial peaks co-occur in individuals whose specific diagnosis is autism, suggesting an increased and more autonomous role of perception in autistic reasoning and intelligence [24].

**Figure 2:**
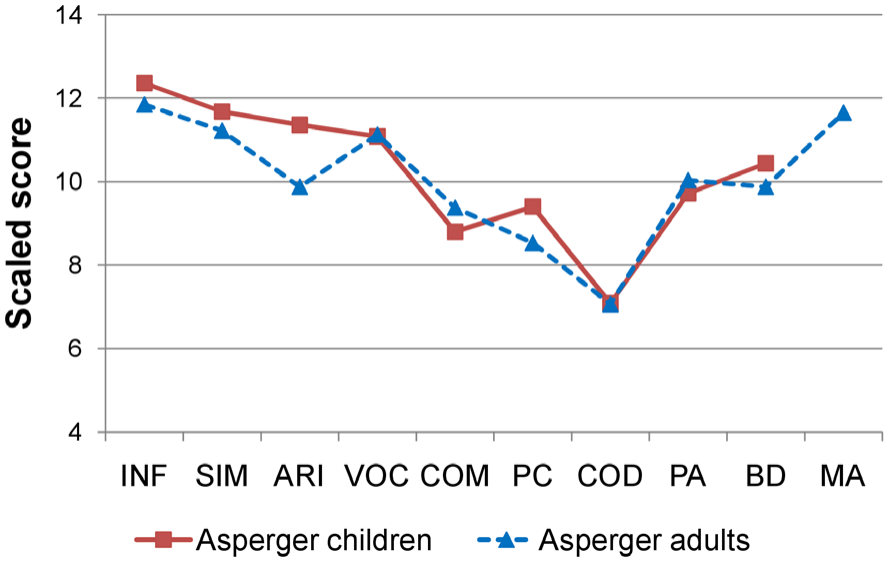
Wechsler subtest profile in Asperger adults and children. Asperger adults are shown in blue and Asperger children in red. INF: Information. SIM: Similarities. ARI: Arithmetic. VOC: Vocabulary. COM: Comprehension. PC: Picture completion. COD: Digit symbol-Coding. PA: Picture arrangement. BD: Block Design. MA: Matrix Reasoning.

But what about individuals whose specific diagnosis is Asperger syndrome? In Dawson et al.'s previous investigations of autistics' RPM performance, Asperger individuals were excluded. Asperger syndrome is a relatively low-prevalence [27] autistic spectrum diagnosis characterized by intelligence scores within the normal range (non-Asperger autistics may have IQs in any range). Two main distinctions between the specific diagnosis of autism and Asperger syndrome are relevant to the question of intelligence in the autistic spectrum. First, while their verbal and nonverbal communication is not necessarily typical across development, Asperger individuals do not, by diagnostic definition, exhibit characteristic autistic delays and anomalies in spoken language. While both autistic and Asperger individuals produce an uneven profile on Wechsler subtests, Asperger individuals' main strengths, in contrast with those of autistics (see [20]), are usually seen in verbal subtests (as illustrated in Figure 2; see also [28]). Although RPM is often deemed a “nonverbal” test of intelligence, in practice typical individuals often rely on verbal abilities to perform most RPM items [29], [30], [31]. Second, at a group level, Asperger individuals do not display the autistic visuospatial peak in Wechsler scales; rather, their Block Design subtest performance tends to be unremarkably equivalent to their FSIQ (see Figure 2 and also [32]). The question of whether Asperger individuals display the autistic advantage on RPM over Wechsler is thus accompanied by the possibility that the Asperger subgroup represents an avenue for further investigating the nature of this discrepancy.

Our goal was to investigate whether the autistic advantage on RPM is also characteristic of Asperger syndrome and, further, whether RPM performance reveals a fundamental property of intelligence across the autistic spectrum. If the mechanism underlying autistics' advantage on RPM is limited to visuospatial peaks or to language difficulties disproportionately hampering Wechsler performance, then the advantage should not be found in Asperger individuals. Indeed, as predicted by Bolte et al. [21], Asperger individuals should perform even better on Wechsler scales than on RPM. If instead the underlying mechanism is more general and versatile, then Asperger individuals should demonstrate at least some advantage on RPM. Preliminary findings have suggested this to be the case. In one recent study, Asperger children (age 6–12) obtained significantly higher raw scores on RPM than did typical children matched on age and Wechsler performance [33].

## Methods

### Participants

#### Asperger participants

The sample included 32 Asperger adults (age 16 to 49 years, *M* = 26.8) and 25 Asperger children (age 7 to 15 years, *M* = 11.9), whose characteristics are summarized in Table 1. The data for the Asperger adults and children were retrieved from the database of the Centre d'excellence en troubles envahissants du développement de l'Université de Montréal. All consecutive individuals who met the diagnostic criteria and had completed both RPM and Wechsler scales (WAIS-III or WISC-III) were entered in the study. Diagnosis was achieved with the ADI-R [34], administered to all participants, complemented by the ADOS (module 3 or 4; [35]) administered to 51 of the 57 participants, as well as clinical expertise. A diagnosis of Asperger syndrome was given if ADI-R scores were above autism thresholds (or a maximum of 2 points under the communication domain threshold) and there was no delayed speech (first single words before 24 months and first phrases before 33 months), echolalia (score of 0, i.e., rarely or never echoes), pronoun reversal (score of 0, i.e., no confusion between first person and second or third person), or stereotyped speech (score of 0 or 1, i.e., speech could be relatively repetitive but not stereotyped in an odd or unusual way), all as measured by the ADI-R. Exclusion criteria were any known genetic or additional neurological conditions.

**Table 1:** Participants' characteristics.

	Children		Adults	
	Asperger	Non-Asperger	Asperger	Non-Asperger
Sample size (gender)	25 (6 F, 19 M)	27 (7 F, 20 M)	32 (4 F, 28 M)	39 (2 F, 37 M)
Age (years)	11.88 (2.62)	11.26 (3.28)	26.84 (9.03)	23.10 (5.03)
Wechsler scales IQ (percentiles)				
FSIQ	52.12 (28.63)	69.26 (20.79)	46.63 (26.88)	68.74 (17.29)
VIQ	63.74 (25.13)	69.78 (20.14)	54.74 (25.83)	67.13 (20.01)
PIQ	41.08 (31.34)	64.78 (23.41)	39.38 (26.44)	64.21 (22.23)
Raven's Progressive Matrices (percentiles)				
	59.05 (30.64)	71.83 (22.41)	67.67 (49.14)	80.66 (18.08)

Numbers are given as Mean (standard deviation). FSIQ: Full-Scale IQ. VIQ: Verbal IQ. PIQ: Performance IQ.

#### Non-Asperger control participants

A sample of 39 adults (age 16 to 37 years, *M* = 23.1) and 27 children (age 6 to 16 years, *M* = 11.3) with typical development was recruited through ads in local newspapers. Exclusion criteria were the presence of personal or familial history of psychiatric, neurological or genetic conditions, as assessed in a semi-structured interview. Some of the control participants were included in a previous study [20].

Informed assent (child participants) and written informed consent (adult participants and parents of child participants) was provided for any data included in the database, which was formally approved by the ethics committee of Rivière-des-Prairies Hospital (Montréal, Canada).

### Tasks and procedure

In an individual setting, all participants completed the standard version of RPM, and child participants completed the Wechsler Intelligence Scale for Children (WISC-III, Canadian norms), whereas adult participants completed the Wechsler Adult Intelligence Scale (WAIS-III, Canadian norms). All instruments were administered by clinicians unaware of the hypotheses of this study. The order of the tests was counterbalanced across participants.

### Data analysis

Non-parametric tests were used for all data analyses. Mann-Whitney U tests were conducted for between-group comparisons of the Wechsler versus RPM difference. Within-group comparisons of Wechsler versus RPM level of performance were carried with Wilcoxon signed-rank tests. Spearman rank correlations were computed separately in each group to assess the presence of associations between RPM performance and Wechsler performance (IQs and subtests). Note that similar results were obtained with parametric tests. All statistical analyses were carried using SPSS 17.

## Results

### Adult samples

**Figure 3:**
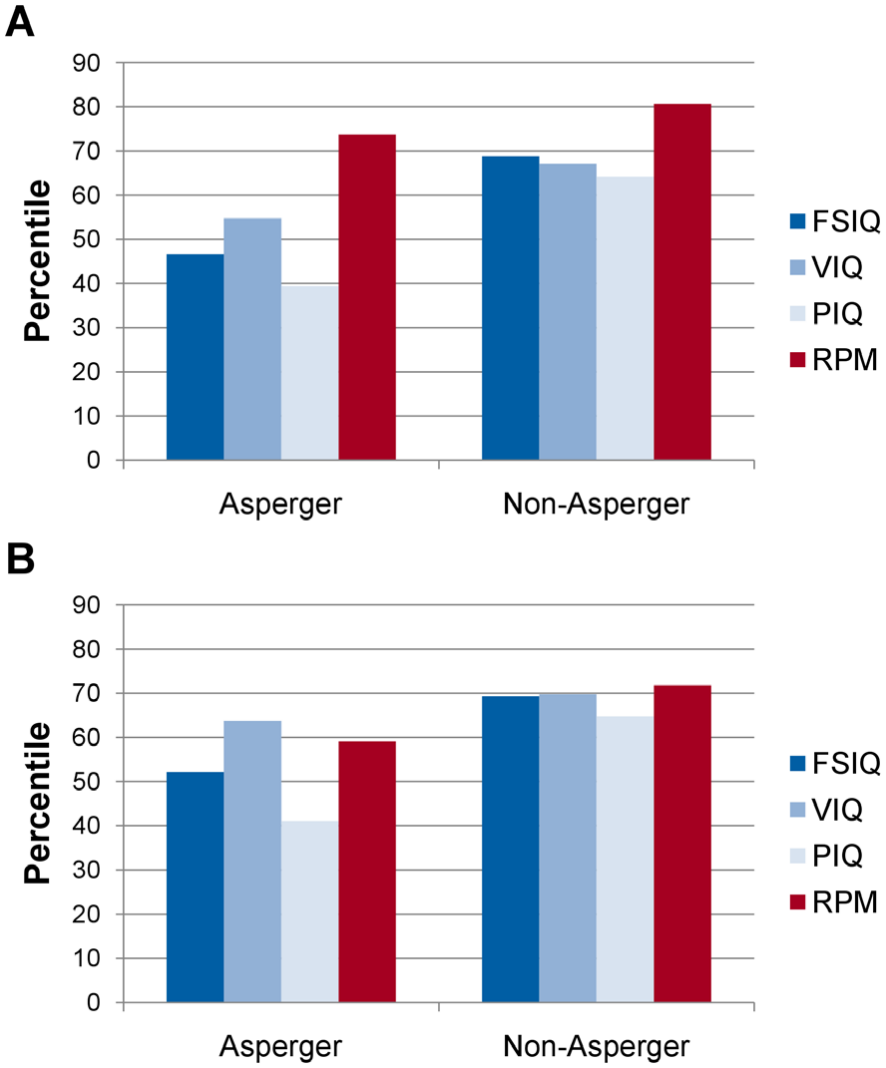
Performance on the Wechsler Intelligence Scales and Raven's Progressive Matrices. Performance on the Wechsler Intelligence Scales (blue) and Raven's Progressive Matrices (red) is shown for A) Asperger adults and non-Asperger adults, and B) Asperger children and non-Asperger children. FSIQ: Full-Scale IQ. VIQ: Verbal IQ. PIQ: Performance IQ.

As illustrated in Figure 3, the average RPM score for Asperger adults was at the 74^th^ percentile, whereas their average Wechsler FSIQ was at 47^th^ percentile (a difference of 27 percentiles). For the non-Asperger adult controls, their average RPM score was at the 81^st^ percentile, whereas their average FSIQ was at the 69^th^ percentile. The Asperger adults demonstrated an advantage of RPM over Wechsler FSIQ that was significantly greater than that of the non-Asperger adult controls, Mann-Whitney *U* = 366.5, *p*<.01.

As often reported in samples of Asperger individuals, the Asperger adults' Wechsler VIQ was significantly higher than their PIQ (55^th^ vs. 39^th^ percentile), *Z* = 3.43, *p*<.01, but the Asperger adults had RPM scores that were significantly higher than both their VIQ and PIQ scores, both *p*s<.01. In contrast, non-Asperger adults had VIQ and PIQ scores that were statistically equivalent (67^th^ vs. 64^th^ percentile), *Z* = 0.61, *p* = .54, and despite their RPM scores exceeding their VIQ and PIQ scores, both *p*s<.01, the magnitude with which their RPM exceeded their PIQ was significantly smaller than it was for the Asperger adults, *U* = 332.0, *p*<.01.

#### Correlations and subtests

Asperger adults' RPM scores were highly correlated with their FSIQ, VIQ, and PIQ scores, *r* = .70, .56, and .80, respectively, all *p*s<.01. Among their Wechsler subtests, the Asperger adults' Matrix Reasoning subtest scores had the highest correlation with their RPM scores, *r* = .71, *p*<.01, and this subtest approached the level of their RPM performance (65^th^ and 74^th^ percentile, respectively). Their performance on three verbal subtests, Information, Similarities, and Vocabulary (66^th^, 62^nd^ and 63^rd^ percentile, respectively; see Figure 2) approached their RPM performance. Their performance on these three subtests was also correlated with their performance on RPM, *r* = .40, .50, and .47, respectively, all *p*s<.05.

For non-Asperger adults, their RPM scores were also correlated with their FSIQ and PIQ scores, *r* = .53 and .47, respectively, *p*s<.01, but no significant correlation was found between RPM and VIQ, *r* = .26, *p* = .11. Among their Wechsler subtests, Matrix Reasoning had the highest correlation with RPM performance, *r* = .63, *p*<.01, as with the Asperger adults.

The difficulty level of each RPM item was computed as the percentage of participants, within each group, who achieved the correct answer. The difficulty level of the 60 items was highly correlated across the Asperger and non-Asperger adults, *r* = .90.

### Child samples

Compared with the RPM-Wechsler discrepancies found for the Asperger adults, the discrepancies found for the Asperger children were less marked. Their average performance was at the 59^th^ percentile on RPM and the 52^nd^ percentile on Wechsler FSIQ. Non-Asperger children obtained almost identical average RPM and FSIQ scores, at the 72^nd^ and 69^th^ percentile. The discrepancy between the two tests was not significantly different in Asperger children and non-Asperger children, *U* = 307.0, *p* = .58.

As with the Asperger adults, there was a significant discrepancy between Asperger children average VIQ (64^th^ percentile) and PIQ score (41^st^ percentile), *Z* = 3.16, *p*<.01, and Asperger children RPM scores were significantly higher than their PIQ, *Z* = 2.64, *p*<.01, but not significantly different from their VIQ scores, *Z* = 1.27, *p* = .21. In contrast, non-Asperger children obtained similar VIQ and PIQ scores (69^th^ and 65^th^ percentile), *p* = .32, and there was no significant difference between the non-Asperger children RPM scores and their VIQ (*p* = .51) or PIQ scores (*p* = .17).

#### Correlations and subtests

For Asperger children, performance on RPM correlated significantly with FSIQ (*r* = .54) and VIQ (*r* = .75) but only marginally with PIQ (*r* = .38, *p* = .06). Three Wechsler verbal subtests—Similarities, Arithmetic and Vocabulary—were the most highly correlated with RPM performance, *r* = .58, .65, and .50, all *p*s≤.01. Asperger children also achieved some of their highest scores on two of these subtests, Similarities and Vocabulary, respectively at the 68^th^ and 61^st^ percentile, above or similar to their RPM performance (59^th^ percentile).

In non-Asperger children, correlation between RPM scores and FSIQ (*r* = .33, *p* = .09) or VIQ (*r* = .36, *p* = .06) approached significance, but there was no significant correlation between RPM and PIQ scores (*r* = .19, *p* = .35). None of their Wechsler subtest scores correlated significantly with their RPM scores.

As with Asperger adults, the difficulty level of the 60 RPM items was highly correlated across Asperger children and non-Asperger children, *r* = .94.

#### Comparisons with autistic children

Data from Asperger children in this study were compared to those of autistic children of a previous study, presented in Figure 1 [20]. Discrepancy between RPM and FSIQ, as well as between RPM and VIQ, was significantly higher in autistic children than in Asperger children, both *p*s<.01. However, the discrepancy between RPM and PIQ did not differ between groups, *p* = .56. Furthermore, although the discrepancy between RPM and Block Design subtest did not differ between the two groups, *p* = .29, the discrepancy between RPM and four other subtests, Information, Vocabulary, Arithmetic, and Similarities, was consistently higher for the autistic than the Asperger children, all *p*s<.05.

For the autistic children, RPM was similarly correlated with FSIQ, VIQ and PIQ, *r* respectively .49, .44 and .51, *p*≤.01, whereas for the Asperger children, RPM was more strongly associated with VIQ than with PIQ. Also, for the autistic children, Block Design was most strongly associated with RPM performance, *r* = .57, *p*<.01, whereas for the Asperger children, the correlation was lower, *r* = .41, *p* = .04. Lastly, for the autistic children, the verbal subtests (Information, Similarities, Arithmetic and Vocabulary) were less strongly associated with RPM, *r* respectively .34, .40, .45 and .35, *p*≤.05, than they were for Asperger children.

## Discussion

Asperger individuals differ from autistics in their early speech development, in having Wechsler scores in the normal range, and in being less likely to be characterized by visuospatial peaks. In this study, Asperger individuals presented with some significant advantages, and no disadvantages, on RPM compared to Wechsler FSIQ, PIQ, and VIQ. Asperger adults demonstrated a significant advantage, relative to their controls, in their RPM scores over their Wechsler FSIQ and PIQ scores, while for Asperger children this advantage was found for their PIQ scores. For both Asperger adults and children and strikingly similar to autistics in a previous study [20], their best Wechsler performances were similar in level to, and therefore plausibly representative of, their general intelligence as measured by RPM.

We have proposed that autistics' cognitive processes function in an atypically independent way, leading to “parallel, non-strategic integration of patterns across multiple levels and scales” [36] and to versatility in cognitive processing [26]. Such “independent thinking” suggests ways in which apparently specific or isolated abilities can co-exist with atypical but flexible, creative, and complex achievements. Across a wide range of tasks, including or perhaps especially in complex tasks, autistics do not experience to the same extent the typical loss or distortion of information that characterizes non-autistics' mandatory hierarchies of processing [24]. Therefore, autistics can maintain more veridical representations (e.g. representations closer to the actual information present in the environment) when performing high level, complex tasks. The current results suggest that such a mechanism is also present in Asperger syndrome and therefore represents a commonality across the autistic spectrum. Given the opportunity, different subgroups of autistics may advantageously apply more independent thinking to different available aspects of information: verbal information, by persons whose specific diagnosis is Asperger's, and perceptual information, by persons whose specific diagnosis is autism.

One could alternatively suggest that the construct measured by RPM is relative and thus would reflect processes other than intelligence in autistic spectrum individuals. However, a very high item difficulty correlation is observed between autistic individuals and typical controls, as well as between Asperger individuals and typical controls. As previously noted [20], these high correlations indicate that RPM is measuring the same construct in autistics and non-autistics, a finding now extended to Asperger syndrome. Therefore, dismissing these RPM findings as not reflecting genuine human intelligence in autistic and Asperger individuals would have the same effect for non-autistic individuals. The discrepancies here revealed between alternative measures of intelligence in a subgroup of individuals underline the ambiguous non-monolithic definition of intelligence. Undoubtedly, autistics' intelligence is atypical and may not be as easily assessed and revealed with standard instruments. But given the essential and unique role that RPM has long held in defining general and fluid intelligence (e.g., [37]), we again suggest that both the level and nature of autistic intelligence have been underestimated. Thus, while there has been a long tradition of pursuing speculated autistic deficits, it is important to consider the possibility of strength-based mechanisms as underlying autistics' atypical but genuine intelligence.
